# Neonatology: First Exposure to Antibiotics from the Ethical Perspective of Parents, Physicians, and Regulators

**DOI:** 10.3390/antibiotics14090936

**Published:** 2025-09-16

**Authors:** Iliya Mangarov, Simeon Iliev, Yulian Voynikov, Valentina Petkova, Iva Parvova, Antoaneta Tsvetkova, Irina Nikolova

**Affiliations:** 1 Department of Neonatology, University Hospital “SofiaMed”, Faculty of Medicine, Sofia University St. Kliment Ohridski, 1504 Sofia, Bulgaria; iliya.mangarov@gmail.com; 2 Independent Researcher, 1680 Sofia, Bulgaria; iliev_simeon@yahoo.com; 3 Department of Chemistry, Faculty of Pharmacy, Medical University, 1000 Sofia, Bulgaria; y_voynikov@pharmfac.mu-sofia.bg; 4 Department of Organization and Economics of Pharmacy, Faculty of Pharmacy, Medical University of Sofia, 1000 Sofia, Bulgaria; vpetkova@pharmfac.mu-sofia.bg; 5 Department of Internal Medicine, Clinic of Rheumatology, St. Anna University Hospital, Medical University of Sofia, 1750 Sofia, Bulgaria; aparvova@medfac.mu-sofia.bg; 6 Medical College, Medical University of Varna, 9002 Varna, Bulgaria; antoaneta.tsvetkova@mu-varna.bg; 7 Department of Pharmacology, Pharmacotherapy and Toxicology, Faculty of Pharmacy, Medical University of Sofia, 1000 Sofia, Bulgaria

**Keywords:** preterm birth, antibiotics, antimicrobial stewardship programs, ethics, parents, antimicrobial resistance

## Abstract

Premature infants are an especially vulnerable group that often needs extended intensive care. Prematurity naturally hampers the development of the immune system, significantly increasing the risk of infections. In the Neonatal Intensive Care Unit (NICU), antibiotic treatment is often a crucial, life-saving measure. For parents, the birth of a very preterm infant (before 32 weeks of gestation) turns what should be a happy event into a period filled with deep uncertainty and distress. Maintaining hope amid these difficulties relies heavily on maintaining regular communication with and trusting the medical team. Clinical realities in the NICU include a high risk of infection that requires multiple medications, including antibiotics. There is an inverse relationship between gestational age and pharmaceutical exposure. Parents worry about the amount of medication their child receives and the potential long-term effects on development. Over the past thirty years, initiatives such as antimicrobial stewardship programs have worked to reduce antibiotic use and treatment duration in the NICU, emphasizing proper care for premature infants worldwide. This article examines the ethical landscape from the perspectives of three primary stakeholders: parents, healthcare providers, and regulatory bodies. The key ethical question is whether these groups achieve meaningful cooperation or if institutional and professional priorities overshadow clinical practice. In the NICU, decision-making responsibility mainly lies with the medical team, as parents often have limited influence over treatment decisions, and regulatory oversight usually occurs indirectly. This concentration of authority underscores the complex and critical nature of neonatal intensive care.

## 1. Introduction

Preterm birth (PTB), defined as delivery before 37 completed gestational weeks, remains the leading global cause of infant mortality and morbidity [1]. Clinically, preterm infants are categorized by gestational age (GA): (1) very preterm infants (VPIs): 28–32 weeks GA, and (2) extremely preterm infants (EPIs): <28 weeks GA. Mortality risk increases exponentially with decreasing GA.

Global epidemiological data reveal a significant PTB burden, with the World Health Organization estimating an incidence of 11.1% (14.9 million births) in 2010 [2]. In 2020, PTB was highest (16.2%) in Bangladesh and lowest (<5.6%) in Finland, Japan, Kazakhstan, Latvia, Lithuania, Norway, Moldova, Serbia, and Sweden [3].

Maternal infections and other pregnancy complications are strongly associated with PTB [4]. PTB directly accounts for nearly one million neonatal deaths annually, representing over 50% of all neonatal mortality worldwide [5]. Geographic disparities are striking, with the highest mortality rates occurring in sub-Saharan Africa and South Asia [5]. The threshold of viability demonstrates significant global variation: (1) in high-income countries, infants born at ≤28 weeks often receive intensive care with survival possible, while (2) in low-resource settings, even infants born at 32 weeks may be considered non-viable. These disparities underscore the critical importance of implementing effective prenatal care policies to reduce PTB incidence and improve outcomes.

Neonates exhibit distinct infection patterns compared to older pediatric patients, with hospital-acquired infections (HAIs), particularly bloodstream infections, being the predominant type [6]. VPIs demonstrate distinct characteristics from near-term infants, including heightened vulnerability to infections [7]. During Neonatal Intensive Care Unit (NICU) hospitalization, preterm infants frequently receive multiple medications, with antibiotic prophylaxis or treatment for suspected/proven early-onset sepsis (EOS) being most common [8,9].

A well-documented inverse correlation exists between GA and the number of drugs per infant, with substantial variability in medication exposure among preterm infants [10]. Systemic antibiotics represent the most frequently administered medications in this population, with the following utilization patterns showing: (1) over 85% of VPIs receive treatment for 7 to 14 days, and (2) more than 95% of EPIs are being treated for an even more extended period. The average hospital stay of VPIs and EPIs is ≥60 days [11,12]. Among antibiotics, penicillins, gentamicin, amikacin, and vancomycin are reported as the most frequently used [12,13,14].

The concern about missing undiagnosed sepsis likely contributes significantly to antibiotic overtreatment in NICUs. Therefore, in a restricted area such as the NICU, clinical decision-making often focuses on when to initiate antibiotic treatment rather than whether to do so. Notably, the majority of antibiotics administered in this setting are used off-label or unlicensed [15,16,17,18,19,20,21]. However, it is essential to note that antibiotics commonly used today have been administered to VPIs for decades, and the NICU’s staff has significant experience with their use. Of greater concern is the introduction of novel or experimental agents, where limited experience creates genuine uncertainty about their effects on this vulnerable population [22,23].

Extended use of early antibiotics without positive blood cultures has faced significant criticism [24]. Most neonates with highly suspected clinical sepsis and negative cultures receive at least 5 days of antimicrobial therapy [24], while those with confirmed meningitis are treated with antibiotics for over 3 weeks [25,26].

Antibiotic exposure in neonates has been consistently linked to delayed microbiota maturation, as well as a reduction in the diversity of bacterial species and strains [27,28,29]. However, longitudinal studies demonstrate that while these disruptions are significant, the gut microbiome shows remarkable resilience, typically achieving functional restoration within 2–3 years post-exposure [30,31].

This study examines ethical considerations surrounding neonatal antibiotic use through three key stakeholder perspectives: parents, healthcare providers, and regulatory bodies, led by the four principles of biomedical ethics (the respect for individuals’ autonomy, the principles of non-maleficence, beneficence, and justice) introduced by Beauchamp and Childress [32]. Our primary research question investigates whether these groups demonstrate collaborative alignment or primarily advocate for their respective interests in clinical decision-making.

## 2. Diagnostic Tools for Initiation, Duration, and Termination of Antibiotic Treatment

Physician decisions to initiate antimicrobial therapy in neonates typically consider three key factors: (1) maternal risk factors, (2) clinical presentation, and (3) laboratory markers [33]. Antimicrobial stewardship programs (ASPs) during the neonatal period should prioritize evidence-based risk assessment while adhering to established clinical protocols. Mukhopadhyay et al. (2019) [34] highlight the importance of stewardship programs for reducing unnecessary antibiotic exposure in preterm infants, particularly those with negative blood cultures.

The Organization for Economic Co-operation and Development (OECD) recognizes ASPs as one of five key interventions offering substantial population health benefits and cost savings. These programs incorporate strategies such as the World Health Organization’s (WHO) Access, Watch, and Reserve (AWaRe) antibiotic classification framework [35]. Despite these initiatives, studies reveal persistent antibiotic prescribing for uninfected preterm neonates, even in facilities with established ASPs [36,37].

Reliable diagnostic tools are crucial for guiding antibiotic decisions in preterm infants, striking a balance between effective infection management and minimizing unnecessary exposure (Table 1). While biomarkers alone lack sufficient specificity to objectively determine the initiation of antibiotics [34], they provide valuable adjunctive information when interpreted in conjunction with clinical findings. Current evidence supports the use of biomarkers as part of a comprehensive assessment to optimize both the duration and discontinuation of antibiotic therapy [38].

The EOS calculator (https://neonatalsepsiscalculator.kaiserpermanente.org; accessed on 1 July 2025) serves as a crucial stewardship tool, helping to reduce unnecessary antibiotic exposure in low-risk, term infants while ensuring prompt treatment for high-risk cases. Designed for infants born at ≥34 weeks’ gestation, this validated risk assessment tool is not recommended for use in very preterm infants (VPIs) [57]. Optimal antibiotic management in preterm neonates requires the following: (1) careful clinical evaluation, (2) culture results, (3) serial inflammatory markers, and (4) adherence to stewardship protocols. Current guidelines recommend early discontinuation when infection becomes unlikely, carefully weighing the risks of undertreatment against potential antibiotic-associated complications [58].

While healthcare providers remain the primary decision-makers in this life-saving process, their daily clinical practice is influenced by multiple stakeholders, including hospital administrators, pharmaceutical companies, and regulatory bodies. Notably, parents of vulnerable neonates often have limited involvement in these critical treatment decisions, despite being profoundly affected by the outcomes.

## 3. The Benefits of Antibiotics

PTB has multiple etiologies, with prenatal infections representing a primary contributor through inflammatory mechanisms. Although antibiotic prophylaxis appears theoretically justified, clinical evidence supporting its efficacy remains limited. A Cochrane review of 14 studies (*n* = 7837 women) found no significant reduction in perinatal or infant mortality with antibiotic treatment for the prevention of infection [59]. While antibiotic therapy for asymptomatic bacteriuria, bacterial vaginosis, Group B Streptococcus (GBS) colonization, and gonorrhea was historically thought to lower PTB risk, contemporary evidence shows minimal or no benefit [60]. Although infection and inflammation are established contributors to PTB, antibiotic prophylaxis lacks conclusive evidence for preventing preterm delivery [61,62]. Recently, Fung and Sahota (2024) [63] demonstrated that antenatal antibiotics before caesarean section or intrapartum prophylaxis ≥ 4 h prior to vaginal delivery may reduce neonatal sepsis risk in term neonates of GBS carriers. Schilling et al. (2022) [64] reported high antibiotic exposure rates (64.6% during pregnancy/childbirth), with 95.3% of PTB cases occurring in antibiotic-exposed women. These findings highlight the critical need for ASPs in perinatal care. Significant research gaps persist regarding antimicrobial strategies during the antepartum/intrapartum periods, as well as comprehensive perinatal approaches [65].

It is worth noting that, despite the documented risks, the dramatic decline in infection-related mortality highlights the critical importance of antibiotic therapy. Manan et al. (2016) [66] demonstrated that timely administration of antibiotics effectively prevents EOS in neonates. Their findings revealed that penicillin plus gentamicin, initiated within 24 h postpartum, provides effective EOS prophylaxis, although treatment failure rates were higher in low-birth-weight infants. The study also noted that VPIs typically required longer treatment courses (7 days) compared to term infants (4–5 days).

Reynolds et al. [67] demonstrated that prophylactic antibiotic administration prior to peripherally inserted central catheter (PICC) line removal reduces the incidence of clinical sepsis in neonates. Their study of 196 infants receiving antibiotics 12 h before percutaneous central catheter removal found significantly lower sepsis rates in the treatment group (2% with vancomycin prophylaxis vs. 13% in untreated controls). These findings indicate that either vancomycin administration before PICC removal or continuation of antibiotics within 12 h after completing a planned course significantly reduces post-removal sepsis risk in preterm infants.

## 4. The Consequence of “Treat One, Damage All”

Antibiotics administered to VPIs aim to prevent or treat confirmed infections (“treat one”). However, they can also have systemic detrimental effects on the developing infant (“damage all”). Prolonged antimicrobial use is associated with dysbiosis and an elevated risk of mortality, severe morbidity, severe bronchopulmonary dysplasia, and necrotizing enterocolitis (NEC). Furthermore, it may lead to long-term adverse health outcomes, including childhood celiac disease, diabetes, and asthma [27,28,29,68,69,70,71,72,73,74,75,76].

Antibiotic treatment in VPIs results in a decreased abundance of beneficial bacteria, such as Bifidobacteria, Bacteroides, and Streptococcus, along with reduced microbial diversity. It also promotes the colonization of pathogenic microorganisms, including Enterobacter, Enterococcus, Clostridium, Staphylococcus, and Escherichia, in the gastrointestinal tract [27,74,77,78]. Infants receiving short-term antibiotic treatment typically have restored gut microbiome diversity within three weeks. In contrast, those undergoing intensive treatment show a sustained reduction in microbial diversity [79].

Invasive candidiasis represents a leading cause of morbidity and mortality in premature infants [74]. Although prematurity itself predisposes infants to candidiasis, the use of broad-spectrum antibiotics remains the most significant modifiable risk factor (Table 2). Antibiotics promote Candida colonization density by diminishing microbial competition within the gut microbiome. Notably, third-generation cephalosporins have explicitly been associated with a two-fold increased risk of candidiasis development [74].

Early-life antibiotic therapy disrupts microbiome development, promotes the carriage of antibiotic resistance genes, and may contribute to rising population-level antibiotic resistance [28,29].

## 5. Medical Professionals’ Attitudes Toward Antibiotic Use in VPIs

*“When VPI is admitted to the NICU, it is intubated, receives central and peripheral lines, and requires umbilical cord nutrition. Daily monitoring involves drawing 1 mL of blood while simultaneously observing its delicate weight fluctuations. In these critical moments, infection risk dominates my clinical concerns—even with negative lab results, I feel compelled to treat. The immediate reality of a distraught mother outside the unit makes abstract considerations about decade-later antibiotic consequences feel secondary. Concerns like asthma and obesity will not even matter if a child doesn’t survive the NICU. While we absolutely recognize the importance of antimicrobial resistance (AMR) as a global health priority, our immediate focus must remain on two fronts: preventing preterm births and maintaining impeccable NICU hygiene to reduce hospital-acquired infections. Please know that every medication decision, including antibiotics, follows rigorous clinical assessment and is never made lightly.”*

Neonatal infections present with nonspecific clinical manifestations that overlap with other common neonatal conditions, including pneumonia, respiratory distress syndrome, metabolic disorders, and intracranial hemorrhage [80]. This diagnostic challenge contributes to wide variations in empirical antibiotic prescribing practices across NICUs, even among institutions with similar patient populations. Current evidence highlights concerning patterns of antibiotic use in VPIs: (1) unnecessary and prolonged antibiotic treatment is documented in numerous studies; (2) research shows serious side effects and suggests long-term adverse health effects in prematurely born infants exposed to antibiotics in early life [81,82,83]; and (3) empirical therapy often continues for 5–7 days despite negative blood cultures [84].

Flannery et al. (2018) [85] demonstrated that the majority of infants, with body weight < 1500 g, from nearly 300 US hospitals, were treated with antibiotics in their first days of life, and approximately 30% of them received >5 days of antibiotic treatment. There were significant differences between hospitals that could not be explained solely by medical reasons. In the period from 2015 to 2018, >50% of infants born at GA < 32 weeks received intravenous antibiotics within the first 14 days of life. The median treatment duration (interquartile range) was 8 (7–10) and 6 (5–7) days for culture-positive and culture-negative EOS, respectively, in the period from 2009 to 2011.

Preterm neonates, remarkably very low birth weight (VLBW) infants born before 32 GA, present unique challenges that limit the applicability of standard pediatric ASP findings. They are often exposed to peripartum risk factors, such as chorioamnionitis, prelabor rupture of membranes, spontaneous preterm labor, or maternal GBS colonization [86]. Additionally, neonates are immunocompromised and tend to deteriorate more rapidly than those born at term. The global death toll from neonatal sepsis is estimated at 400,000 annually, with over 30% of survivors experiencing long-term neurodevelopmental issues [87,88,89]. The perceived risks of delaying treatment lead to the early use of broad-spectrum empirical antibiotics in neonatal care [90]. Moreover, the definitions of certain neonatal infections, such as ventilator-associated pneumonia and urinary tract infections, are inconsistent, and no standardized treatment protocols have been established in the literature (Table 3).

VPIs represent an exceptionally vulnerable patient population requiring prolonged intensive care. Their premature birth results in an immunologically underdeveloped state, significantly increasing susceptibility to infections [91]. While antibiotic therapy in the NICU is frequently essential and life-saving, infections in this population originate from two primary routes: (1) vertical transmission [(maternal–fetal), e.g., ESBL (extended-spectrum beta-lactamase producers) E. coli, MRSA (Methicillin-resistant Staphylococcus aureus), CRE (Carbapenem-resistant Enterobacteriaceae)] or (2) horizontal transmission [hospital-acquired infections (HAIs), e.g., MRSA, VRE (Vancomycin-resistant Enterococci), CRE, ESBL)]. This epidemiology underscores the dual necessity of comprehensive prenatal monitoring and rigorous infection control measures in NICUs.

Neonatal bacterial infections frequently lack pathogen identification, necessitating broad-spectrum antibiotic therapy for suspected sepsis. VPIs receive antibiotics significantly more often than term infants (~80% vs. <10%), reflecting several key factors, like heightened infection risk (e.g., sepsis, pneumonia), frequent empiric treatment initiation, prolonged NICU hospitalization, nonspecific clinical presentation overlapping with sepsis, and the lack of definitive “gold standard” in diagnosing neonatal sepsis [92].

The management of potential sepsis differs between mature and premature infants [93]. While validated online tools like the Kaiser calculator are available for infants born at ≥34 weeks GA [8,94,95], no similar tool exists for VPIs. However, protocolized approaches, such as those described by Kitano et al. (2019) [96], have proven effective in reducing unnecessary antibiotic use through systematic assessment of maternal and neonatal clinical status, the infant’s sepsis risk score, hemoculture results, and symptom progression over time. “Clinical status” and “progression of symptoms” in VPIs are particularly challenging aspects of such tools, both regarding when to start treatment and how long to continue it. The accuracy of a clinician’s assessment depends on more than just skill; close monitoring and multiple physical exams can decrease unnecessary antibiotic exposure [97], but this requires sufficient human and material resources. Furthermore, collaboration with obstetricians is crucial for providing accurate information about the circumstances of preterm birth and for evaluating risk factors. Access to efficient laboratory services (including rapid pathogen identification and resistance profiling) influences decision-making [98,99]. These varying resources and capabilities contribute to differences in practice across NICUs. Clinicians face the ongoing challenge of balancing the risk of untreated infection against the potential short- and long-term adverse effects of antibiotic exposure. Institutional clinical studies can offer evidence-based guidance to reduce diagnostic uncertainty and help optimize antibiotic use while ensuring patient safety [100].

## 6. Parents’ Attitude Towards Decision-Making Regarding Antibiotic Treatment

The power dynamics surrounding antibiotic decision-making in the NICU present a paradox: while infants and their parents bear the greatest stakes in outcomes, they often hold the least decision-making authority [101,102]. Achieving accurate shared decision-making in this setting necessitates a fundamental restructuring of communication practices, decision-making processes, and power relationships [99,101,103]. A critical first step is establishing information symmetry, ensuring that parents not only receive medical information but also fully comprehend it through precise language, visual aids, and confirmation of understanding [104,105]. Clinicians must move beyond merely conveying facts to foster meaningful dialogue about uncertainties, risks, and family preferences, requiring dedicated training in advanced communication skills [101,103].

Parents consistently express a need for (1) daily clinical updates, (2) clear interpretation of biomarkers and culture results, and (3) discussion of alternative approaches, such as a “watch-and-wait” strategy [99,103]. Their attitudes toward antibiotic use are shaped by a complex interplay of emotional, cultural, and informational factors. Many experience dual anxiety, fearing both untreated infections and potential treatment side effects [104]. While most parents accept antibiotic therapy when risks and benefits are transparently communicated, ongoing dialogue is essential to address concerns and sustain trust [84,105]. Clinicians report that parents typically defer to medical judgment when infants exhibit acute symptoms (e.g., respiratory distress, lethargy), prioritizing immediate infection risks over long-term considerations, such as antimicrobial resistance (AMR) [84,105]. However, persistent knowledge gaps complicate the process of shared decision-making. Although parents generally recognize antibiotics’ role in treating bacterial infections, awareness of AMR and treatment specifics (e.g., indications, duration, side effects) remains limited [106,107,108,109]. Many families underestimate personal AMR risks, perceiving themselves as “low users” of antibiotics [105]. Despite these gaps, physician recommendations have a significant influence on parental confidence in treatment safety [75,110].

To integrate families into the decision-making process, structured opportunities for discussion must be prioritized—even in time-sensitive scenarios [97,100]. While actual emergencies demand immediate action, many NICU antibiotic decisions allow for brief but meaningful family consultation. Implementing protected pause points (e.g., 5–10 min for deliberation) can balance clinical efficiency with parental involvement [99,101]. Additionally, cultural competence is vital; care teams must recognize how cultural backgrounds shape perceptions of medical authority, risk tolerance, and family roles in decision-making [106,107,108,109]. Some families may defer entirely to clinicians, while others expect active collaboration—both approaches warrant respect and accommodation [75,110].

## 7. The Attitude of Regulatory Authorities

In recent decades, regulatory agencies have become increasingly concerned about the rising global threat of AMR [111,112]. AMR has arisen from a combination of natural selection and human activities, mainly the misuse and overuse of antimicrobials in human and veterinary medicine, as well as agricultural practices [100]. The evolutionary process allows resistant pathogens to develop effective survival strategies and spread rapidly across borders through international travel, food distribution networks, and human migration patterns [113,114]. Additionally, environmental contamination occurs through pharmaceutical waste, agricultural runoff containing antimicrobials, and hospital effluent that carries resistant organisms and genes [115].

The European Union/European Economic Area (EU/EEA) reports over 670,000 annual antibiotic-resistant bacterial infections, resulting in approximately 33,000 deaths [116]. AMR is now recognized by the WHO as one of the top ten global health threats [117], with about 1.27 million deaths directly linked to drug-resistant infections worldwide in 2019. GBD 2021 Research by Antimicrobial Resistance Collaborators [118] estimated 1.28 million deaths due to AMR in 2030, and it could reach 1.91 million globally by 2050. From 2025 to 2050, the combined forecast predicts 39.1 million deaths attributable to AMR and 169 million deaths associated with AMR [117]. These alarming figures confirm that AMR is a critical public health challenge of the 21st century [118]. Effective mitigation requires a coordinated, multisectoral effort to address pathogen evolution, resistance reservoirs, and transmission routes, forming effective containment strategies. However, the complex interactions among AMR drivers across different ecosystems continue to challenge our understanding of its epidemiology [111].

Similar to other populations, antibiotic use in neonates carries both short-term and long-term risks, particularly through the development of AMR, which affects individual patients and the entire NICU by altering local resistance patterns, changing unit epidemiology, and reducing treatment effectiveness for future patients.

To address antibiotic overuse in NICUs, regulatory agencies, healthcare institutions, and quality improvement initiatives have put in place structured ASPs. These programs focus on evidence-based prescribing, robust monitoring systems, and prescriber accountability to ensure antibiotics are used only when necessary. Healthcare professionals share responsibility for fighting AMR through stewardship [119]. ASPs are behavioral interventions that require hospitals to have multidisciplinary teams, including infectious disease (ID) specialists, clinical pharmacists, and microbiologists, to reduce AMR and hospital costs. ASPs encourage careful antimicrobial use and can extend the effective lifespan of these drugs, while new antibiotics for pediatrics are being approved [99,120,121]. They have proven success in adult and pediatric populations by optimizing antimicrobial use, prolonging antibiotic effectiveness, and reducing healthcare costs. Nonetheless, NICUs face unique challenges for traditional ASP approaches. Issues stem from nonspecific clinical signs, limited evidence on treatment durations for many pediatric infections, a shortage of pediatric ID specialists, and the complexities of laboratory testing and interpretation [122].

A systematic review on the effect of ASP in NICUs was conducted by da Silva et al. (2020) [123]. The authors identified five studies [84,124,125,126,127], and a reduction in broad-spectrum antibiotic use was reported in four of them [84,124,125,126]. Ting et al. (2019) [127] reported no improvement in the inappropriate use of antibiotics in VPIs. However, no study evaluated the impact on AMR, which is a long and multifactorial process, and we hardly expect ASPs to have any significant impact on AMR in the human population in the near future. Limiting the use of broad-spectrum antibiotics and shortening the duration of antibiotic treatment will affect the individual patient and the hospital by decreasing HAIs. Overall, implementing ASPs in NICUs leads to reduced unnecessary antibiotic use. Mascarenhas et al. (2024) [128], in a meta-analysis involving 70 studies and over 350,000 neonates, conclude that moderate-to-low-certainty evidence indicates that neonatal ASP interventions are associated with a reduction in the initiation and duration of antimicrobial use, without increasing mortality or the reinitiation of antimicrobial agents.

The growing challenge of antimicrobial-resistant pathogens has driven expanded adoption of ASPs in clinical practice [34,73,129,130,131]. In preterm neonates, ASPs primarily aim to minimize empirical antibiotic administration in the immediate postnatal period and optimize treatment duration through risk-stratified approaches. Although some studies suggest a shorter course (3–4 days) may be sufficient, overall evidence and clinical guidelines favor longer durations (5–7 days) due to the risks associated with incomplete treatment [44,73,99,132,133].

While ASPs mainly involve clinical staff, successful implementation depends on leadership understanding clinical complexities, promoting transparency, a non-punitive culture, and providing institutional support [134,135]. Nzegwu et al. (2017) [126] demonstrated the importance of collaborative efforts between public health authorities and clinicians. Their NICU-specific ASP model adapted the Centers for Disease Control and Prevention (CDC) “Get Smart for Healthcare” framework and evidence-based guidelines by Patel & Saiman (2012) [136]. In the studies reviewed, general efforts to reduce antibiotic use at the hospital level were less effective in decreasing antibiotic exposure in premature infants; however, they did improve the profile of antibiotics used. This suggests that a locally tailored, multifaceted, and comprehensive approach, particularly one that incorporates individual patient outcome measures, is preferred.

Mascarenhas et al. (2024) [128] conducted a comprehensive systematic review (70 studies) and meta-analysis (33 studies) evaluating antimicrobial stewardship programs (ASPs) in neonatal populations (both preterm and term infants across NICU and postnatal ward settings). Their analysis examined three key outcomes—clinical outcomes, cost-effectiveness, and antimicrobial resistance. Key findings from the VLBW subgroup analysis (five studies [97,137,138,139]) included a significant reduction in antimicrobial initiation rates in the post-ASP group (80.5%) compared to the pre-ASP group (91.9%) [97,138,139,140]. The authors emphasize that ASP implementation effectively reduces unnecessary antimicrobial exposure in neonates while maintaining patient safety [128].

## 8. The Role of the Pharmaceutical Industry

The NICU setting presents unique pharmacological challenges characterized by a high prevalence of polypharmacy, frequent use of off-label/unlicensed medications, increased risk of adverse drug reactions (ADRs), medication errors, and developmental complications [141,142,143,144,145]. Despite decades of clinical use, most medications lack formal dosing guidelines and approved indications for VPIs. Since antibiotics are used in over 80% of VPIs, who are also very underweight and fragile, there are many hurdles to conducting clinical research. Pharmaceutical companies lack incentives to develop drugs and dosing guidelines for premature infants due to economic (concerns about cost-effectiveness), ethical (parental reluctance to enroll critically ill neonates), and physiological (limited blood volume that restricts sampling) issues [114,146,147,148,149]. Thus, data on the neonatal population are currently limited, with few clinical trials involving neonates to evaluate the efficacy, safety, and dosing of new antibiotics. The new neonatal labeling updates lag significantly behind those for pediatrics. Recently, Bradley et al. (2025) [150] provided supportive evidence for the safety of approved ceftazidime/avibactam doses in premature neonates (≥ 26 GA) for treating suspected or confirmed Gram-negative bacterial infections.

This analysis identifies a critical research gap: What economic and regulatory incentives would effectively motivate pharmaceutical companies to pursue formal drug approval processes for preterm neonates? Current stakeholder dynamics reveal a persistent status quo:

- Parents prioritize immediate neonatal survival over long-term pharmacologic outcomes.
- Clinicians focus on acute clinical management within existing therapeutic paradigms.
- Pharmaceutical companies maintain current developmental pipelines based on market incentives.
- Regulatory agencies continue post-marketing surveillance and data aggregation.

A critical analysis suggests that specific research approaches may require reconsideration. Conducting traditional clinical trials in extremely low-birth-weight (<1 kg) neonates presents substantial methodological and ethical challenges. While we do not advocate for additional conventional clinical studies in this vulnerable population, we emphasize the critical need for (1) comprehensive NICU data collection, (2) systematic observational studies, and (3) registry-based research. These approaches will generate essential evidence to inform future therapeutic strategies for preterm neonates across European healthcare systems.

The widespread use of off-label and unlicensed medications in very preterm infants (VPIs) raises critical questions about the sufficiency of existing evidence. Despite decades of clinical application across numerous NICUs globally, fundamental uncertainties persist regarding pharmacokinetics, optimal dosing, and long-term safety in this vulnerable population. While some argue that real-world experience should suffice for regulatory approval, the scarcity of controlled clinical trials in VPIs [151,152,153,154,155,156] limits robust conclusions on efficacy and safety. Notably, evidence from neonatal pharmacotherapy suggests that a small number of well-designed trials can yield definitive recommendations in favor of particular interventions, whereas the majority (≈90%) of studies either remain inconclusive or contradict initial hypotheses [156,157,158,159].

Given the extensive antibiotic utilization data accumulated over the past decade in European NICUs, each major city typically houses at least one tertiary center, and there exists an opportunity to construct a large-scale, real-world evidence database. Such a repository could provide regulators with the necessary pharmacovigilance and outcome data to formalize indications for preterm infants, bridging the gap between clinical practice and evidence-based approval [160]. Moving forward, leveraging existing observational datasets alongside targeted prospective studies may offer a pragmatic pathway to refining neonatal drug labeling without necessitating large, logistically challenging clinical trials.

## 9. The Interdependent Systems of Neonatal Intensive Care

**Figure 1:**
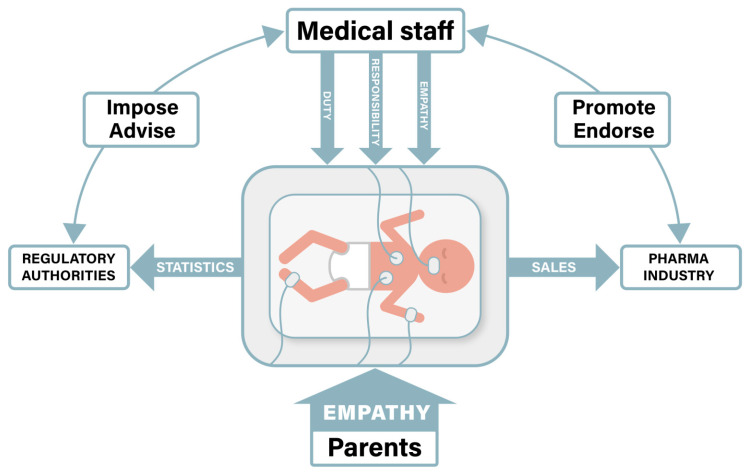
Participants involved in saving a premature life.

The survival and developmental outcomes of critically ill neonates are fundamentally dependent upon three interdependent systems: (1) the clinical expertise of NICU staff, (2) regulatory frameworks governing neonatal care, and (3) pharmaceutical innovation (Figure 1). This tripartite structure currently operates with limited parental agency, as infection control protocols often restrict physical interaction between parents and infants to mitigate HAI risks. Consequently, families lack meaningful mechanisms to actively participate in therapeutic decision-making or neurodevelopmental support during this critical period. The ethical implications of this exclusion warrant careful consideration, particularly in relation to how risk-benefit calculations prioritize institutional infection control measures over familial bonding opportunities that may confer both psychological and developmental benefits.

Parents, despite being the individuals who will bear the long-term consequences of early medical decisions, often find themselves in the outer sphere, receiving information rather than actively participating in decisions [101,104,105]. Their vulnerability is compounded by emotional distress, lack of medical knowledge, and the intimidating NICU environment [102]. Healthcare providers occupy the middle sphere, holding immediate decision-making authority but constrained by institutional protocols, time pressures, and the need to make rapid decisions with incomplete information [32,81,87,109]. While they possess the technical knowledge and clinical authority, they also experience vulnerability through moral distress when forced to make decisions that may conflict with their professional judgment or personal values [131,132]. Regulatory bodies and institutional administrators occupy the outer sphere, influencing practice through policies, guidelines, and resource allocation but remaining removed from individual clinical decisions [35,112,114,161]. Their power is indirect but pervasive, shaping the environment within which clinical decisions are made through antimicrobial stewardship programs, formulary restrictions, and quality metrics [123,129,136].

Among the key stakeholders influencing neonatal outcomes, medical staff and parents are fundamentally aligned in prioritizing infant wellbeing. Optimizing these relationships requires (1) adequate staffing ratios, (2) specialized training in developmental care for healthcare providers, and (3) expanded parental access protocols that balance infection control with bonding opportunities. In contrast, as illustrated in Figure 1, regulatory bodies and pharmaceutical companies operate through indirect mechanisms, primarily collecting aggregate data rather than engaging in direct patient care. These latter stakeholders demonstrate inherent tensions in their operational priorities. The pharmaceutical industry’s growth model depends on sales revenue, creating disincentives for practices that might reduce medication utilization. For instance, antibiotic stewardship programs, while clinically warranted, directly conflict with profit motives, potentially driving companies toward promoting higher-margin alternatives [146]. This economic reality, combined with complex regulatory pathways, helps explain the persistent lack of approved indications for neonatal populations. The substantial investments required for formal drug registration in preterm infants are further discouraged by perceived market limitations and protracted approval processes, creating a systemic barrier to evidence-based pharmacotherapy in this vulnerable population.

In this context, the complexity of pharmacotherapy in preterm infants presents significant challenges, with no clear solutions. Systemically administered medications exert whole-body effects—a pharmacological principle that becomes critically consequential when treating patients weighing ≤ 1 kg. While extensive literature documents off-label and unlicensed drug use in pediatric populations, most studies aggregate data across heterogeneous age groups, obscuring the unique pharmacodynamic and pharmacokinetic considerations required for preterm neonates. For this vulnerable population, each day of development carries profound implications for survival and long-term outcomes. Although neonatal care continues to advance through clinical experience and technological innovation, there remains an urgent need to strengthen evidence-based medicine (EBM) in neonatology. Current NICU practices worldwide often rely heavily on institutional traditions and individual clinician experience [14,114,162]. A paradigm shift toward integrating three core EBM principles is essential: (1) the best available research evidence, (2) clinical expertise, and (3) patient-centered values that recognize neonates as future contributors to society (Table 4) [163]. This approach would help reconcile the tension between the immediate demands of life-saving interventions and the long-term imperative to optimize neurodevelopmental and metabolic outcomes.

## 10. Conclusions

Our research employs a multidimensional ethical framework to examine the complex interplay of stakeholders involved in preterm infant care. At its core, this framework acknowledges that while the medical team’s expertise remains paramount for neonatal survival, wielding primary authority over treatment protocols, parents retain specific rights, including the right to refuse treatment and make discharge decisions, alongside an entitlement to transparent communication about their infant’s condition [101]. The prolonged NICU stay often precipitates profound psychological distress for parents, who typically navigate stages of self-blame, grief, helplessness, and eventual acceptance [102].

### 10.1. Ethical Framework for Decision-Making in NICUs

Four foundational bioethical principles guide this analysis. Autonomy, though central to medical ethics, assumes complexity in the NICU, where parents serve as surrogate decision-makers for non-verbal infants [104,105]. Beneficence obligates clinicians to promote infant wellbeing, yet defining “benefit” for extremely preterm neonates involves navigating uncertainties between immediate survival and long-term morbidity [34,99]. The principle of non-maleficence faces particular tension in antibiotic decisions, where both administration (risking dysbiosis/resistance) and withholding (potentially enabling lethal infections) carry significant harms [27,28,29,68,69,70,71,72,73,75,76]. Justice extends beyond individual cases to encompass resource allocation, equitable access to NICU care, and the societal implications of AMR [112,116,117,118,161].

These principles intersect with three additional ethical considerations salient to neonatology. The Best Interest Standard, although theoretically straightforward, fractures in practice as stakeholders prioritize competing values: parents emphasize survival and comfort, clinicians weigh short-term and long-term outcomes, and public health officials consider population-level AMR consequences [80,84,93,105]. Vulnerability ethics underscores neonates’ unique susceptibility—they cannot advocate for themselves, possess minimal physiological reserve, and face lifelong ramifications from early interventions [5,87,88,89]. Finally, while shared decision-making represents the ideal model, its implementation in the NICU must address inherent barriers, including time constraints, profound emotional distress, and persistent information asymmetry [99,101,103].

Stakeholder interpretations of “best interest” reveal fundamental divergences. Parents, immersed in the emotional immediacy of their infant’s critical state, often prioritize survival and symptom relief, perceiving antibiotic risks as abstract relative to acute infection threats [75,84,104,105,110]. Clinicians, drawing on their cumulative experience, balance immediate needs against long-term sequelae, such as NEC or fungal sepsis—a perspective that frequently generates moral distress when confronting stewardship responsibilities [79,81,83,106,119,134,135,136]. Regulatory and public health entities, meanwhile, assess best interest through a population health lens, emphasizing AMR mitigation and equitable resource distribution—priorities that may conflict with bedside imperatives [95,112,116,117,118,161,174].

### 10.2. Antimicrobial Stewardship

Regulatory and pharmaceutical ecosystems indirectly shape NICU practices through medication availability and AMR policies. ASPs, which have been implemented over the past three decades, have achieved ~25% reductions in NICU antibiotic use, yielding three key benefits: (1) improved neonatal outcomes, (2) decreased healthcare costs, and (3) reduced transmission of resistant pathogens [114,146]. Maximizing ASP efficacy requires integration with multimodal infection prevention (hand hygiene, sterilization protocols, staff vaccination, and visitor policies), demonstrating superior results to standalone stewardship [95]. Unit-specific antibiograms further optimize therapy by enabling narrower-spectrum regimens tailored to local resistance patterns [175].

### 10.3. Systemic Solutions

Effective AMR mitigation demands a systems-based approach addressing five critical domains: (1) enhanced antenatal monitoring and preterm birth prevention [176]; (2) rigorous NICU infection control [166,177]; (3) continuous staff education on diagnostics and prevention [178]; (4) development of rapid pathogen identification technologies [42,179]; and (5) pharmaceutical investment in neonatal antibiotic development [180]. When implemented cohesively, these strategies offer dual benefits: immediate clinical improvements and progressive AMR containment [181]. Sustainable progress, however, will require unprecedented institutional commitment, policy reform, and elevation of neonatal health as a global public health priority.

In conclusion, the care of preterm infants in the NICU presents a profound ethical and clinical challenge, requiring careful navigation of competing priorities among stakeholders (clinicians, parents, and public health systems). While medical expertise must guide life-saving decisions, actual ethical practice demands meaningful parental engagement, transparent communication, and respect for the complex emotional journey families endure. The principles of autonomy, beneficence, non-maleficence, and justice provide a foundational framework; however, their application remains fraught with tension, particularly in antibiotic decision-making, where immediate risks and long-term consequences must be carefully weighed.

Ultimately, optimizing care for preterm infants is not merely a medical or ethical imperative but a societal one (Table 5). By aligning clinical expertise with family values, stewardship responsibilities, and systemic reforms, we can uphold the best interests of these vulnerable patients while safeguarding the future of effective antibiotic therapy.

**Future Perspectives:** The current evidence base regarding therapeutic interventions for VPIs remains limited, highlighting the urgent need for comprehensive data on treatment regimens, duration, survival outcomes, and long-term developmental effects. Significant heterogeneity exists across studies regarding ASP implementations, patient populations, and outcome measures, emphasizing the need for standardized reporting frameworks to facilitate meaningful comparisons [176,182]. We introduce the innovative concept of pregnancy–antibiotic–neonate stewardship programs (PAN-SPs) as an integrated approach to manage care from pregnancy through the neonatal period. By targeting modifiable risk factors during pregnancy and optimizing antibiotic use in affected neonates, these programs may decrease both preterm birth rates and antimicrobial exposure, potentially reducing the development of AMR [183,184]. Establishing consensus guidelines for reporting patient-centered outcomes in ASP research is crucial for advancing this field, as it would improve the interpretation of intervention effectiveness [114]. Additionally, ASP protocols should be tailored specifically for neonatal populations, since the reasons for and drivers of antimicrobial use differ fundamentally from those in term infants. Sustainable progress will depend on coordinated national efforts involving regulatory agencies and healthcare systems to implement evidence-based practices and monitor their influence on antibiotic prescribing and resistance patterns.

ASPs have demonstrated measurable success in reducing unnecessary antibiotic use, but their full potential depends on integration with broader infection control strategies and culturally sensitive, family-centered care. To achieve sustainable progress, a multilevel framework must be implemented across clinical, institutional, and public health domains. At the clinical level, this involves enhancing shared decision-making through improved clinician–parent communication, establishing structured opportunities for deliberation during critical decision-making points, and providing ongoing education about the risks and benefits of antibiotics. Institutionally, ASPs should be expanded to incorporate unit-specific antibiograms, multimodal infection prevention bundles, and dedicated support systems for healthcare teams navigating complex ethical dilemmas. From a public health perspective, neonatal health must be prioritized within broader antimicrobial resistance mitigation strategies through targeted investments in rapid diagnostic technologies and advocacy for equitable access to specialized NICU care. Concurrent research efforts should focus on accelerating the development of neonatal-specific antimicrobial agents and investigating the long-term consequences of early-life antibiotic exposure, ensuring stewardship practices evolve alongside our understanding of their developmental impacts. This integrated approach addresses both immediate clinical needs and the systemic challenges of antimicrobial resistance in vulnerable neonatal populations.

**Limitations:** This review has several significant limitations that should be noted. First, our analysis focused specifically on VPIs born before 32 weeks’ GA, which may limit how well our findings apply to other neonatal populations. Second, we did not consider birth weight in our assessment, despite its well-established correlation with GA and clinical outcomes. Third, our review was limited to antibiotic use and did not include other pharmacotherapies commonly used in the NICU. Notably, this manuscript incorporates qualitative perspectives from both family and clinical stakeholders, including the lived experiences of relatives of a VPI born at 30 weeks’ gestation (Y.V. and I.N.) and the professional insights of the attending neonatologist (I.M.). While these personal accounts offer valuable context, they represent individual experiences that may not reflect broader population trends. Future research would benefit from more comprehensive systematic reviews that incorporate diverse gestational ages, birth weight parameters, and a full range of neonatal pharmacotherapies.

## Figures and Tables

**Table 1:** Tools for initiation, duration, and termination of antibiotic treatment in VPIs.

Tools for the Initiation of Antibiotics (Highly Subjective and Nonspecific)		
**Maternal Risk Factors:** Chorioamnionitis, preterm prelabor rupture of membranes, spontaneous onset of preterm labor, or maternal Group B *Streptococcus* colonization.		
**Blood Culture** (gold standard for confirmation): Limited use as a basis for initial empirical antibiotic use [39]. It is desirable to take two samples from different sites, especially if CNS infection is suspected, as well as samples in aerobic and anaerobic environments [26]. Should be collected before starting antibiotics (at least 1 mL of blood). Time-consuming—takes up to 72 h.		
**Lumbar Puncture (if sepsis/meningitis suspected)**: Indicated in symptomatic infants or cases of positive blood culture.		
**Gastric aspirate, tracheal aspirate** in intubated infants. **Urine culture** when CNS infection is suspected. **Peripheral secretions**: Ear (up to 12 h after birth), anal, nasal, throat, and, if symptoms are present, eye, umbilical, and skin lesions (pustules, etc.).		
**Molecular Diagnostics (PCR, Multiplex Panels)**: Rapid pathogen detection (e.g., 16S rRNA PCR, sepsis panels)		
**Unspecific makers with low predictive values**	**Clinical Signs and Symptoms**	Respiratory distress, apnea, temperature instability, cold extremities, feeding intolerance, tachycardia, cyanosis, hypotension, poor peripheral perfusion [26].
	**Full Blood Count**	Abnormal white blood cell count: leukocytosis, leukopenia (more sensitive marker): up to 24 h < 8000 and >30,000; up to 72 h < 5000 and >20,000; after 72 h > 15,000, neutropenia, or an elevated immature-to-total neutrophil (I/T) ratio: in early infections > 0.2; in late infections > 0.14, red cell distribution width to platelet ratio (RPR) [40,41]
	**Procalcitonin (PCT)**	Early marker (within 3–6 h of infection) for bacterial infection (demonstrating higher specificity than CRP for bacterial infections). Serial measurements (0 h/24 h/48 h) to guide decisions; expensive [42,43,44,45]. Values depend on GA. Physiological increase in PCT during the first days of life. Cut-off values: Low risk: <0.5. Intermediate risk: 0.5–2. High risk: >2.
	**C-Reactive Protein (CRP)**	Rise 12–24 h after infection and peak at 48–72 h. Lower specificity. Serial measurements (0 h/24 h/48 h) to guide decisions [42,43,46].
	**Interleukin-6 (IL-6)**	The concentration of IL-6 rises rapidly after the onset of bacteremia, but its half-life is short. Serum IL-6 has high accuracy in detecting neonatal sepsis [47,48,49]. Cut-off values: First day of life < 80 pg/mL. Second to seventh day of life < 40 pg/mL. Seventh day of life < 30 pg/mL.
	**Presepsin**	The optimal timing for presepsin measurement and whether presepsin may be helpful before the onset of symptoms remains to be determined [50,51,52,53].
	**Progranulin**	A promising biomarker for the diagnosis of EOS [54].
**Tools for Duration of Antibiotic Treatment**		
**Serial CRP/PCT Trends**		Declining levels suggest a response to therapy. PCT declines faster with treatment, making it valuable for stewardship.
**Blood Culture Results** (gold standard for confirmation)		Serves as the basis for transitioning from empiric treatment to a continued course of therapy and can be used to guide rational antibiotic selection [47,55]. If negative at 36 to 48 h, reevaluate the necessity of ongoing antibiotics.
**Clinical Improvement**		Resolution of symptoms (e.g., apnea, feeding tolerance).
**Tools for Termination of Antibiotic Treatment**		
**Negative Blood Cultures (≥48–72 h)**		The most common reason to stop is if the cultures are negative and the infant is improving [56].
**Normalizing Inflammatory Markers (CRP and PCT)**		Have good negative predictive accuracy to rule out sepsis within 24 to 48 h: CRP < 10 and PCT < 0.5 ng/mL) [46].
**Clinical Stability**		No ongoing signs of infection.

Abbreviations: CRP: C-reactive protein; EOS: early-onset sepsis; PCR: polymerase chain reaction; PCT: procalcitonin.

**Table 2:** Commonly used antibiotics in newborns.

Antibiotics	Doses Used in Neonates ^1^
**Penicillins**	Used in staphylococcal infections, excluding Methicillin-resistant staphylococci
Benzylpenicillin (Penicillin G)	**Empiric treatment of EOS** **(in combination with an aminoglycoside)** 30–90 mg/kg/dose; every 6–8 h 60 mg = 100,000 units of penicillin
	400,000 U/kg/24 h, every 8 h in GBS meningitis
	50,000 U/kg/24 h every 8–12 h for 10 days in high probability of congenital syphilis
Oxacillin	50–100 mg/kg/24 h every 8–12 h ^1^
Nafcillin	25 mg/kg/24 h every 8–12 h ^1^
**Broad-spectrum penicillins**	Used in a wide range of bacteria, including both Gram-positive and Gram-negative bacteria
Ampichhillin	**Empiric treatment of suspected EOS including meningitis (with an aminoglycoside)** 50–100 mg/kg/24 every 8–12 h
	300 mg/kg/24 in meningitis ^1^
Carbenicillin	200–400 mg/kg/24 h in 3–4 applications ^1^
Azlocillin	100–200 mg/kg/24 h ^1^
Piperacillin	100 mg/kg/24 h in 2–3 applications ^1^
Amoxicillin	**Empiric treatment of suspected EOS including meningitis (with an aminoglycoside)** 50 mg/kg, every 8–12 h
	100 mg/kg every 8–12 h for meningitis
Amoxicillin/Clavulanic acid	60 mg/kg/24 h every 12 h ^1^
Ampicillin/Sulbactam	75–150 mg/kg/24 h every 12 h ^1^
Piperacillin/Tazobactam	80–100 mg/kg dose every 6–8 h Effective in HAI: Acinetobacter, Klebsiella, Pseudomonas, incl. ESBL
**Aminoglycosides**	**Empiric treatment of suspected EOS (in combination)**
Gentamicin	4–5 mg/kg every 36–48 h
Amikacin	7.5 mg/kg/12 h ^1^ Used for suspected or proven Gram-negative infection resistant to other aminoglycosides 12–14 mg/kg every 36–48 h
Tobramhycin	4–5 mg/kg/dose every 24–36–48 h depending on current body weight
**Cephalosporins**	Cephalosporins are not first-line antibiotics when initiating routine empirical AB therapy after birth due to the rapid development of resistance to them. In newborns, mainly third- and fourth-generation cephalosporins are used, and they are used empirically as a third-line antibiotic in severe infections or after isolation of the bacterial strain according to the antibiogram.
Cefazolin (I generation)	25–50 mg/kg/dose every 12 h
Cefuroxime (II generation)	40–100 mg/kg/24 h every 12 h ^1^
Ceftazidime (III generation)	60–100 mg/kg/24 h every 12 h ^1^ 50 mg/kg/dose every 8–12 h suitable for Gr (-) meningitis, pronounced anti-pseudomonal activity
Cefotaxime (III generation)	50 mg/kg/dose every 8–12 h
Ceftriaxone (III generation)	50–75 mg/kg/24 h One application, not recommended in hyperbilirubinemia (displaces bilirubin from its binding to albumin). Alternative therapy for gonococcal infection and congenital syphilis.
Cefepime (IV generation)	30 mg/kg every 12 h
Cefoperazone/Sulbactam	60–80 mg/kg/24 h in 2–3 applications ^1^
**Glycopeptides**	Crucial in the fight against Gram-positive pathogens
Vancomycin	10–15 mg/kg/24 h once or 2–3–4 applications depending on body weight and day after birth for Gr (+) bacteria 15 mg/kg every 8–12–18 h depending on cGA
**Lincosamides**	In the neonatal period, they are mainly used for osteoarthritis, with a course of treatment lasting 21 days.
Lincomycin	10 mg/kg/24 h
Clindamycin	5 mg/kg/dose every 8 h
**Macrolides**	Administered orally, which limits their use in newborns in serious condition
Clarithromycin	7.5 mg/kg/24, every 12 h orally
Azithromycin	10–20 mg/kg/dose daily IV for 3 days Eradication of *Ureaplasma urealyticum* in preterm infants
Flucloxacillin	25–50–100 mg/kg/dose every 8–12 h
**Carbapenems**	They are used for Gr (-) microorganisms, Klebsiella, Serratia, Enterobacter
Imipenem	15 mg/kg/24 every 8 h
Meropenem	20–40 mg/kg/dose every 8–12 h The dose depends on GFR
**Fluoroquinolones**	Active against *Staph. aureus*, *Streptococcus*, and Gr (-) microorganisms, including *Pseudomonas*. Due to reports of growth disorders, they are used in the neonatal period only for vital indications and in the presence of sensitivity (antibiogram).
Ciprofloxacin	6–8 mg/kg/24, every 12 h
Levofloxacin	6–8 mg/kg/24, once or every 12 h
**Polymyxins**	Effective against multidrug-resistant *Pseudomonas aeruginosa*, *Klebsiella pneumoniae*, *Acinetobacter*, and β-lactamase multidrug-resistant *Enterobacteriaceae*
Polymyxin E	50,000–150,000 IU/kg/day every 8 h in infusion for 30 min to 1 h
**Systemic antimycotics**	**Empiric treatment of suspected or confirmed invasive fungal infections**
Fluconozole	Initial dose 6–12 mg/kg, then 3–6 mg/kg every 72–24 h
**Antiprotozoal medication**	Used to treat certain bacterial and parasitic infections, including NEC in neonates
Metronidazole	Loading dose IV or oral 15 mg/kg, Maintenance dose IV or oral 7.5 mg/kg/dose every 12–24 h

^1^ The dose and regimen of most antibiotics depend on factors such as gestational age and weight. Abbreviations: AB: antibiotic; cGA: corrected gestational age; EOS: early-onset sepsis; ESBL: extended-spectrum beta-lactamase; GBS: Group B Streptococcus; GFR: glomerular filtration rate; HAI: hospital-acquired infection; NEC: necrotizing enterocolitis.

**Table 3:** Recommendations for the choice and duration of antibiotic therapy.

Microorganism	Antibiotic	Bacteremia	Meningitis
GBS	Ampicillin or Ampicillin/Sulbactam or Benzylpenicillin	10 days	14–21 days
*Escherichia coli*	Cefotaxime or Ampicillin + Gentamicin or Amicacin	10–14 days	21 days
CoNS	Vancomycin	7 days	14 days
*Klebsiella*, *Serratia*	Cefotaxime or Meropenem + Gentamicin or Amikacin	10–14 days	21 days
*Enterobacter*, *Citrobacter*	Cefepime or Meropenem + Gentamicin	10–14 days	21 days
*Enterococcus*	Ampicillin or Vancomycin + Gentamicin	10 days	14–21 days
*Listeria*	Ampicillin + Gentamicin	10–14 days	14–21 days
*Pseudomonas*	Ceftazidime or Piperacillin/Tazobactam + Gentamicin or Tobramycin	14 days	21 days
MSSA	Nafcillin or Meticillin	10–14 days	21 days
MRSA	Vancomycin	10–14 days	22 days

Abbreviations: CoNS: Coagulase-negative staphylococci; GBS: Group B Streptococcus; MSSA: Methicillin-susceptible Staphylococcus aureus; MRSA: Methicillin-resistant Staphylococcus aureus.

**Table 4:** Evidence-based approaches to AMR in neonates.

Category	Strategy	Impact on AMR	Key Evidence
**Rational AB use**	Narrow-spectrum empiric therapy (e.g., ampicillin + gentamicin) for EOS. De-escalate or stop if culture is negative at 48–72 h.	Reduces selection pressure for resistant strains.	[164,165,166]
**Duration of AB therapy**	Short-course (5–7 days) for uncomplicated sepsis. Avoid prolonged empiric therapy.	Decreases risk of resistance and dysbiosis; equivalent efficacy with shorter courses.	[55,81,138,139]
**ASPs**	NICUs adopt protocols for empiric therapy.	Reduces unnecessary broad-spectrum use.	[81,123,131,167]
**Infection Prevention**	Strict ward and personnel hygiene. Maternal GBS prophylaxis.	Lowers infection rates, reducing the need for ABs.	[168,169,170]
**Probiotics**	*Lactobacillus*/*Bifidobacterium* for preterm neonates.	Decreases antibiotic exposure; reduces NEC/LOS.	[169]
**AMR Surveillance**	Monitor NICU pathogens (e.g., ESBL *E. coli*, CRE, MRSA). NICU-specific resistance patterns should guide therapy. Use rapid diagnostics (PCR).	Guides targeted therapy, avoids empiric overuse.	[164,171,172,173]
**Global Policies**	WHO “AWaRe” classification for neonates.	Standardizes rational AB use.	[35]

Abbreviations: AB: antibiotic; AMR: antimicrobial resistance; ASPs: antimicrobial stewardship programs; CRE: carbapenem-resistant Enterobacteriaceae; EOS: early-onset sepsis; ESBL: extended-spectrum beta-lactamase; MRSA: methicillin-resistant Staphylococcus aureus; NICU: neonatal intensive care unit; NEC: necrotizing enterocolitis/; LOS: late-onset sepsis; PCR: polymerase chain reaction; WHO “AWaRe”: WHO access, watch, and reserve.

**Table 5:** Summary of ethical principles for NICU decision-making by stakeholders.

Ethical Principle/Consideration	Parents	Physicians/Clinicians	Regulators and Public Health Officials
Autonomy	Act as surrogate decision-makers for their non-verbal infant. Their role is to represent the infant’s and family’s interests and values.	Must respect parental autonomy (as surrogates) by providing full information and involving them in the shared decision-making process.	Prioritize procedural justice and system-wide policies that ensure equitable access to care, often superseding individual autonomy.
Beneficence	Often interpret “benefit” as immediate survival and symptom relief for their critically ill infant.	Obligated to promote infant wellbeing by balancing immediate life-saving needs against potential long-term sequelae (e.g., NEC, dysbiosis).	Define benefit through a population health lens, focusing on broad outcomes like AMR containment and equitable resource distribution.
Non-Maleficence	Perceive the risk of withholding antibiotics (leading to infection) as a more concrete and immediate harm than the abstract risk of future dysbiosis or AMR.	Face acute tension: both administering (risking dysbiosis/resistance) and withholding (risking lethal infection) antibiotics carry significant potential harms.	Focus on mitigating systemic harm, primarily the long-term public health threat of antimicrobial resistance (AMR).
Justice	Focus is almost exclusively on justice for their individual child and securing the best possible care and resources for them.	Focus on micro-allocation of resources and treatments at the unit or patient level.	Focus on macro-allocation of resources, equitable access to care, and the societal implications of AMR.
Best Interest Standard	Prioritize survival and comfort, viewing antibiotic risks as secondary to the acute threat of infection.	Weigh short-term vs. long-term outcomes, often experiencing moral distress when stewardship goals conflict with individual patient concerns.	Assess best interest through population-level outcomes, which may conflict with bedside imperatives (e.g., restricting antibiotic use to curb AMR).
Vulnerability	Experience vulnerability due to emotional distress, lack of medical knowledge, and an intimidating NICU environment.	Experience vulnerability through moral distress when constrained by protocols or forced to make decisions that conflict with their clinical judgment.	Not directly vulnerable in the clinical context, but their policies must be designed to protect the vulnerable (neonates and families).
Shared Decision-Making	Desire to actively participate but face barriers like information asymmetry, emotional distress, and time constraints.	Hold the responsibility to facilitate shared decision-making by bridging information gaps and creating space for parental values amidst time pressures.	Their policies (e.g., ASPs, guidelines) create the framework and constraints within which bedside decisions are made.
Primary Ethical Tension	The immediacy of their infant’s critical condition vs. the abstract, long-term risks of treatment (e.g., dysbiosis, AMR).	The duty to act in the best interest of the immediate patient vs. the responsibility to practice stewardship for the broader community’s health.	The mandate to protect public health and resources at a population level vs. supporting individualized care at the bedside.

## Abbreviations

The following abbreviations are used in this manuscript:

ADRs	Adverse Drug Reactions
AMR	Antimicrobial Resistance
ASPs	Antimicrobial Stewardship Programs
AWaRe	Access, Watch, and Reserve classification of antibiotics
CDC	Centers for Disease Control and Prevention
cGA	Corrected Gestational Age
CoNS	Coagulase-Negative Staphylococci
CRE	Carbapenem-Resistant Enterobacteriaceae
CRP	C-Reactive Protein
EBM	Evidence-Based Medicine
EOS	Early-Onset Sepsis
EPIs	Extreme Preterm Infants
ESBL	Extended-Spectrum Beta-Lactamase Producers
GA	Gestational Age
GBS	Group B Streptococcus
HAIs	Hospital-Acquired Infections
ID	Infectious Disease
IL-6	Interleukin-6
I/T ratio	Immature-to-Total Neutrophil Ratio
MRSA	Methicillin-Resistant Staphylococcus aureus
MSSA	Methicillin-Susceptible Staphylococcus aureus
NEC	Necrotizing Enterocolitis
NICU	Neonatal Intensive Care Unit
OECD	Organization for Economic Co-operation and Development
PAN-SPs	Pregnancy–Antibiotic–Neonate Stewardship Programs
PCT	Procalcitonin
PICC	Percutaneously Inserted Central Catheter
RPR	Red Cell Distribution Width to Platelet Ratio
PTB	Preterm Birth
VPIs	Very Preterm Infants
VLBW	Very Low Birth Weight
VRE	Vancomycin-Resistant Enterococci
WHO	World Health Organization
